# Application value of blood-brain barrier and peripheral inflammatory markers in early diagnosis of pancreatic encephalopathy in severe acute pancreatitis

**DOI:** 10.3389/fnins.2026.1930294

**Published:** 2026-08-11

**Authors:** Lin Xu, Chao Liu, Guochao Zhu

**Affiliations:** 1 Department of Hepatobiliary Surgery, Affiliated Hospital of Jianghan University/The Sixth Hospital of Wuhan, Wuhan, Hubei, China; 2 Department of Intensive Care Medicine, Affiliated Hospital of Jianghan University/The Sixth Hospital of Wuhan, Wuhan, Hubei, China

**Keywords:** blood-brain barrier, early diagnosis, interleukin-6, matrix metalloproteinase-9, monocyte chemoattractant protein-1, pancreatic encephalopathy, S100β protein, severe acute pancreatitis

## Abstract

**Objective:**

To investigate the value of blood-brain barrier (BBB) injury markers [S100β protein, matrix metalloproteinase-9 (MMP-9), cerebrospinal fluid/serum albumin ratio (QAlb)] and peripheral inflammatory markers [tumor necrosis factor-α (TNF-α), monocyte chemoattractant protein-1 (MCP-1), interleukin-6 (IL-6)] in the early diagnosis of severe acute pancreatitis (SAP)-related pancreatic encephalopathy (PE).

**Methods:**

A prospective single-center observational cohort study was conducted. A total of 282 SAP patients admitted from March 2024 to February 2026 were consecutively enrolled, with an Acute Physiology and Chronic Health Evaluation II (APACHE II) score ≥ 8, or a CT Severity Index (CTSI) score ≥ 7, or persistent organ failure > 48 h. Patients with pre-existing central nervous system diseases, traumatic brain injury, intracranial infection, alcohol dependence, Wernicke’s encephalopathy, severe liver or kidney diseases, or pregnancy and lactation were excluded. Venous blood samples were collected within 24 h after admission (D0) and on day 3 (D3), day 7 (D7), and day 14 (D14) of the disease course. Serum levels of BBB injury markers (S100β and MMP-9) and peripheral inflammatory markers (TNF-α, IL-6, and MCP-1) were measured by enzyme-linked immunosorbent assay (ELISA). Among the 282 enrolled patients, 50 (17.73%) who met clinical indications and provided informed consent underwent lumbar puncture for QAlb measurement. The primary outcome was the occurrence of PE during hospitalization. Differences in each indicator between the PE and non-PE groups at each time point were compared. Generalized estimating equations (GEE) were used to analyze the temporal trajectories of each indicator. Pearson correlation analysis was performed to examine correlations among indicators. The bootstrap method was used to test the mediating effects. Receiver operating characteristic (ROC) curves were constructed to evaluate the diagnostic performance of individual markers and combined models.

**Results:**

Among the 282 SAP patients, the incidence of PE was 21.99% (62/282). In the PE group, TNF-α, IL-6, and MCP-1 levels were significantly higher than those in the non-PE group at D0 (*P* < 0.05), peaked at D3, and remained significantly elevated at D7 and D14 (*P* < 0.05). In the PE group, MMP-9 and S100β showed no significant differences from the non-PE group at D0 (*P* > 0.05), but began to increase significantly from D3, remained at high levels at D7, and declined at D14 while still remaining significantly higher than those in the non-PE group (*P* < 0.01). Among the 50 patients who underwent lumbar puncture, 22 were in the PE group and 28 in the non-PE group; the QAlb level at D3 in the PE group was significantly higher than that in the non-PE group (*P* < 0.05). GEE analysis of temporal trajectories showed that TNF-α, IL-6, and MCP-1 were significantly elevated at D3 and D7 compared with D0, and significantly decreased at D14 compared with D0. MMP-9 and S100β were significantly elevated at D3, D7, and D14 compared with D0 (*P* < 0.05). Pearson correlation analysis revealed a strong positive correlation between S100β and MMP-9 (*r* = 0.742, *P* < 0.01). Bootstrap mediation analysis confirmed that IL-6 and MCP-1 exerted significant indirect effects on the occurrence of PE through the MMP-9/S100β axis, with mediation effect sizes of 0.336 and 0.312, accounting for 74.50 and 72.90% of the total effects, respectively, while the direct effects were not significant (*P* > 0.05). ROC analysis showed that the area under the curve (AUC) of the combined model of MMP-9 + S100β + MCP-1 reached 0.901, which was significantly superior to single markers and other combined models. The length of hospital stay and 28-day mortality in the PE group were both higher than those in the non-PE group (*P* < 0.05).

**Conclusion:**

The combined model of MMP-9 + S100β + MCP-1 can be used in clinical practice to identify high-risk PE patients at an early stage. Moreover, this study verified the causal chain of inflammation-induced BBB damage leading to brain injury in PE, providing a theoretical basis for MMP-9-targeted prevention strategies for PE.

## 1 Introduction

Acute pancreatitis (AP) is a type of inflammatory disease of the pancreas. Approximately 20% of patients will progress to severe acute pancreatitis (SAP) (He et al., 2025). Pancreatic encephalopathy (PE) is one of the serious neurological complications of SAP. Studies have shown that the combined incidence of PE in AP patients is approximately 11%, and the mortality rate can be as high as 43%. Multi-organ failure (OR > 5) is one of the significant risk factors for death in PE patients, greatly threatening their quality of life and survival prognosis (Meng et al., 2023). However, due to the lack of specificity in the clinical manifestations of PE and the difficulty in early identification, treatment is often initiated too late for optimal efficacy once the condition is recognized, resulting in poor prognosis. Therefore, finding rapid and effective biomarkers to achieve early diagnosis of SAP-related PE is crucial for improving the neurological functional outcome and overall prognosis of patients.

The pathogenesis of PE involves multiple factors such as systemic inflammation and disruption of the blood-brain barrier (BBB) (Dai et al., 2026). Relevant studies have indicated that cytokines such as tumor necrosis factor-α (TNF-α), monocyte chemoattractant protein-1 (MCP-1), and interleukin-6 (IL-6) are closely related to the occurrence and development of SAP (Greer et al., 2022). Farrell PR et al. further demonstrated that MCP-1 participates in the recruitment of white blood cells in AP, thereby exacerbating the acute local tissue damage of SAP; at the same time, IL-6 also has significant value in predicting the severe evolution of the disease in SAP (Farrell et al., 2021). This suggests that peripheral blood inflammatory indicators may have a potential association with the occurrence of SAP-related PE. At the BBB disruption level, matrix metalloproteinase-9 (MMP-9) not only can cleave the vascular basement membrane and intercellular tight junction proteins to enhance BBB permeability, but also can promote the infiltration of inflammatory cells into the central nervous system, thereby inducing neuronal and synaptic dysfunction in various diseases (Song et al., 2022). In addition, S100β, compared with other brain injury markers, can more specifically reflect changes in BBB integrity rather than neuronal damage itself (Liu et al., 2024); the cerebrospinal fluid/serum albumin ratio (QAlb), as a quantitative indicator of BBB permeability, is also widely used to assess the functional status of the central nervous system barrier (Bruno et al., 2024). However, most current studies on peripheral inflammatory markers and BBB markers have mainly focused on SAP. The systematic application of these markers in the early diagnosis of pancreatic encephalopathy is still relatively rare. Therefore, this study, using a prospective single-center observational cohort design, aims to investigate the value of BBB injury markers and peripheral inflammatory markers in the early diagnosis of SAP-related PE, thereby providing objective evidence to facilitate early risk stratification, guide timely targeted interventions, and ultimately enhance both neurological recovery and overall survival in PE patients.

## 2 Materials and methods

### 2.1 General information

From March 2024 to February 2026, a total of 282 patients with SAP were consecutively enrolled in this study.

Inclusion criteria: ➀ Compliant with the SAP diagnostic criteria (Banks et al., 2013); ➁ Acute Physiology and Chronic Health Evaluation II (APACHE II) (LeGall et al., 1986) ≥ 8 points, or CT Severity Index (CTSI) (Balthazar et al., 1990) ≥ 7 points, or persistent organ failure > 48 hours; ➂ Age ≥ 18 years; ➃ Individuals or their legal guardians who provided written informed consent.

Exclusion criteria: ➀ Pre-existing diagnosis of central nervous system diseases; ➁ Complicated with traumatic brain injury or intracranial space-occupying lesions; ➂ Alcohol dependence or Wernicke’s encephalopathy; ➃ Complicated with other severe liver or kidney diseases or malignant tumors; ➄ Pregnant or lactating women, or patients with mental disorders; ➅ Use of medications affecting levels of inflammatory factors or MMP-9 prior to enrollment; ➆ Recurrent or chronic pancreatitis with acute exacerbation. This study was approved by the hospital’s ethics review committee (approval number: WHSHIRB-K-001).

Sample size: According to the literature, the combined incidence of pancreatic encephalopathy in patients with SAP is approximately 11% (Meng et al., 2023). With a two-sided significance level of α = 0.05 and a margin of error δ = 0.04, using the single proportion formula $n=Z_{1-\alpha /2}^{2}\times p\times \left(1-p\right)/\delta^{2}$, substituting *p* = 0.11, the required sample size was approximately 235 cases. Considering a 10% dropout rate, the final plan was to include at least 261 cases. This study actually enrolled 282 SAP patients, with 62 cases (21.99%) in the PE group, meeting the requirements for statistical analysis.

### 2.2 Methods

#### 2.2.1 Observational indicators

Blood specimens (approximately 4–6 mL) were obtained from peripheral veins at admission within the first 24 hours (D0) and again on D3, D7, and D14 of the clinical course. All samples were anticoagulated and then centrifuged at 3,000 r/min for a duration of 10 min. The blood samples were centrifuged within 2 h, and the serum was separated and stored at −80°C until analysis. The levels of S100β, IL-6, TNF-α, MMP-9, and MCP-1 were determined by ELISA.

#### 2.2.2 QAlb measurement

For SAP patients who provided informed consent and met the indications for lumbar puncture, a single lumbar puncture was performed on D3. A 3 mL sample of CSF was collected via aseptic lumbar puncture, along with paired blood specimens. The CSF samples were centrifuged at 3,000 rpm for 5 min, and the supernatant was collected. The levels of albumin in serum and cerebrospinal fluid were determined using the Roche e411 fully automatic electrochemiluminescence analyzer (Roche, Germany) and the rate turbidimetry method. The QAlb value was calculated based on the ratio of cerebrospinal fluid albumin to serum albumin.

It should be noted that lumbar puncture is not a routine examination and was performed only when clinically indicated. In this study, the indications for lumbar puncture included: (1) uncertain diagnosis of pancreatic encephalopathy (PE) requiring differentiation from central nervous system infections (e.g., meningitis, encephalitis); (2) the need to rule out significantly elevated intracranial pressure or neurological emergencies such as subarachnoid hemorrhage; and (3) severe impairment of consciousness (Glasgow Coma Scale score ≤ 12) or new-onset focal neurological signs, necessitating cerebrospinal fluid (CSF) analysis to assist in etiological clarification. All lumbar punctures were performed after obtaining informed consent from the patients or their legal representatives, and were conducted by experienced neurologists under strict aseptic conditions. It is important to emphasize that lumbar puncture and QAlb measurement were performed solely based on clinical diagnostic needs rather than being mandatory requirements of the study protocol. QAlb data were derived only from the subgroup of patients (*n* = 50) who consented to lumbar puncture and met the clinical indications. This subgroup was used exclusively to supplement the assessment of blood-brain barrier permeability differences between PE and non-PE patients and did not constitute the primary outcome measure; therefore, it did not affect the main conclusions of the overall cohort study. Comparison between the 50 patients who underwent lumbar puncture and the 232 patients who did not revealed no statistically significant differences in age, sex, APACHE II score, CTSI score, or PE incidence between the two groups (all *P* > 0.05), indicating that this subgroup was well representative and that the risk of selection bias was low.

#### 2.2.3 Primary outcome and grouping

The primary outcome of this study was the occurrence of pancreatic encephalopathy (PE) during hospitalization. The diagnosis of PE was independently established by two neurologists, with any disagreements adjudicated by a senior neurologist. The diagnostic criteria for PE required that all of the following conditions be met simultaneously (Johnson and Tong, 1977): ➀ New-onset neuropsychiatric symptoms occurring during the course of severe acute pancreatitis, including but not limited to: Disturbance of consciousness (manifested as inhibitory symptoms such as lethargy, somnolence, or coma); Mental and behavioral abnormalities (manifested as excitatory symptoms such as delirium, agitation, hallucinations, or disorientation); Seizures or other focal neurological deficits. ➁ Exclusion of encephalopathy caused by other etiologies through comprehensive clinical and laboratory evaluations, including: Metabolic encephalopathies (e.g., hepatic encephalopathy, uremic encephalopathy, diabetic ketoacidosis, electrolyte disturbances); Pulmonary encephalopathy or hypoxic encephalopathy; Drug-induced encephalopathy or sedative withdrawal reactions; Wernicke’s encephalopathy (excluded by medical history and/or vitamin B_1_ level testing); Central nervous system infections (excluded by cerebrospinal fluid analysis in patients who underwent lumbar puncture). ➂ Cranial CT or MRI performed during the episode of neuropsychiatric symptoms to rule out organic intracranial lesions, including: Intracranial hemorrhage or cerebral infarction; Intracranial space-occupying lesions (tumors, abscesses, etc.); Traumatic brain injury. Patients who met all of the above criteria were diagnosed with pancreatic encephalopathy. Patients were divided into the PE group (*n* = 62) and the non-PE group (*n* = 220) based on whether they developed PE during hospitalization.

### 2.3 Quality control

All clinical data were collected using uniformly designed case report forms. Data were independently entered by two individuals and cross-verified.

### 2.4 Statistical analysis

The data were processed using SPSS 26.0 and R 4.2.1. The measurement data were evaluated for normality using the Shapiro-Wilk test. Data that met the normal distribution criteria were expressed as mean ± standard deviation (x ± s), and comparisons between groups were performed using the independent sample *t*-test. Count data were presented as the number of cases (%), and comparisons between groups were conducted using the χ^2^ test or Fisher’s exact probability method, as appropriate. Repeated measurement data at multiple time points were analyzed using generalized estimating equations (GEE). Pearson correlation analysis was performed to examine the correlations between indicators. The significance of the mediating effect was tested using the bootstrap method (repeated sampling 5,000 times). The MMP-9 + S100β + MCP-1 combined model was validated using the bootstrap internal validation method (repeated sampling 1,000 times). At the same time, 10-fold cross-validation was used to evaluate the generalization performance of the model. Two-sided tests were conducted, and a *P* < 0.05 was considered statistically significant.

## 3 Results

### 3.1 Patient disposition and study flow

**FIGURE 1:**
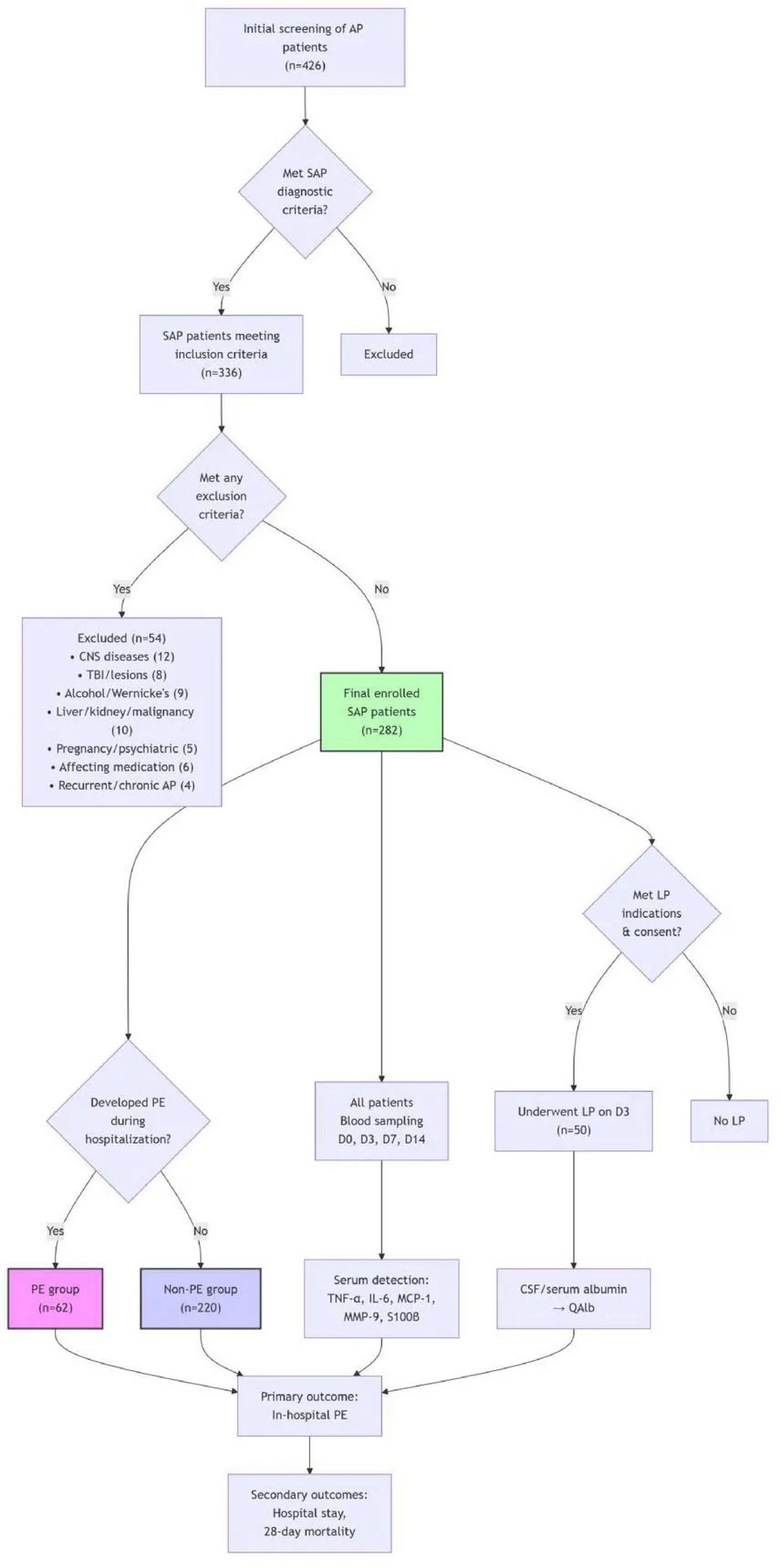
Research flowchart.

A total of 426 patients with acute pancreatitis were initially screened, of whom 336 met the diagnostic criteria for SAP After excluding 54 patients (12 with pre-existing central nervous system diseases, 8 with traumatic brain injury or intracranial space-occupying lesions, 9 with alcohol dependence or Wernicke’s encephalopathy, 10 with severe liver/kidney diseases or malignancies, 5 who were pregnant/lactating or had mental disorders, 6 with medication history affecting inflammatory markers, and 4 with recurrent or chronic pancreatitis with acute exacerbation), 282 SAP patients were finally enrolled. Among them, 62 cases (21.99%) were diagnosed with pancreatic encephalopathy (PE) during hospitalization and were assigned to the PE group, while the remaining 220 cases (78.01%) without PE were assigned to the non-PE group. Of the 282 patients, 50 (22 in the PE group and 28 in the non-PE group) consented to lumbar puncture due to clinical diagnostic needs and underwent QAlb measurement on D3. The study flowchart is presented in Figure 1.

### 3.2 Comparison of general information

Among the 282 patients with SAP in this study, 62 cases were diagnosed with pancreatic encephalopathy (PE), with an incidence rate of 21.99%. There were no significant differences in age, BMI, or gender between the PE group and the non-PE group (all P > 0.05). The PE group had higher APACHE II scores (20.37 ± 4.59), higher CTSI scores (8.25 ± 2.03), and a higher proportion of patients with persistent organ failure > 48 h (61.29%) compared with the non-PE group (all *P* < 0.05) (see Table 1).

**TABLE 1:** Comparison of general information between the two groups [$\bar{x}\pm$ s, n(%)].

Characteristic		PE group (*n* = 62)	Non-PE group (*n* = 220)	*t/χ* ^2^	*P*-value
Age (years)		46.37 ± 11.79	45.61 ± 10.95	0.475	0.636
BMI (kg/m^2^)		23.53 ± 3.42	24.67 ± 4.56	1.828	0.069
Sex, male		33 (53.23)	110 (50.00)	0.201	0.654
Etiology	Biliary	33 (53.23)	110 (50.00)	1.028	0.795
	Hyperlipidemic	13 (20.97)	43 (19.55)		
	Alcoholic	8 (12.90)	37 (16.82)		
	Idiopathic	8 (12.90)	30 (13.64)		
APACHE II score		20.37 ± 4.59	15.39 ± 3.88	8.562	< 0.001
CTSI score		8.25 ± 2.03	6.01 ± 1.18	11.052	< 0.001
Persistent organ failure > 48 h		38 (61.29)	72 (32.73)	16.586	< 0.001

### 3.3 Intergroup comparison of peripheral inflammatory markers

At D0, TNF-α, MCP-1, and IL-6 levels in the PE group exceeded those in the non-PE group, reaching their peak at D3 and remaining markedly elevated through D7 and D14 (all *P* < 0.05; Table 2).

**TABLE 2:** Comparison of peripheral inflammatory marker levels between the two groups ($\bar{x}\pm$ s).

Marker	Time point	PE group (*n* = 62)	Non-PE group (*n* = 220)	*t*	*P*
TNF-α (pg/mL)	D0	39.11 ± 8.42	20.25 ± 4.32	23.931	<0.001
	D3	59.01 ± 11.25	38.79 ± 8.72	15.072	<0.001
	D7	45.82 ± 9.13	27.28 ± 6.15	18.661	<0.001
	D14	36.44 ± 7.63	15.38 ± 3.71	30.247	< 0.001
IL-6 (pg/mL)	D0	106.21 ± 22.46	92.42 ± 20.57	4.568	<0.001
	D3	188.54 ± 42.22	122.53 ± 29.64	13.999	<0.001
	D7	142.33 ± 31.19	115.86 ± 27.84	6.436	<0.001
	D14	112.46 ± 25.12	72.35 ± 13.47	16.689	< 0.001
MCP-1 (ng/L)	D0	44.28 ± 8.23	35.17 ± 7.45	8.307	<0.001
	D3	66.74 ± 8.59	55.45 ± 10.74	7.616	<0.001
	D7	60.41 ± 14.45	49.86 ± 10.08	6.564	<0.001
	D14	34.17 ± 6.53	28.56 ± 5.22	7.053	< 0.001

### 3.4 Comparison of blood-brain barrier injury markers

In the PE group, MMP-9 and S100β showed no significant differences from the non-PE group at D0 (*P* > 0.05). From D3 onward, MMP-9 and S100β in the PE group increased, remained at high levels at D7, and declined at D14 while still remaining higher than those in the non-PE group (all *P* < 0.05).

Lumbar puncture was performed on 50 consenting patients (22 from the PE group and 28 from the non-PE group). At D3, the PE group exhibited a higher QAlb than the non-PE group (*P* < 0.05) (see Tables 3, 4).

**TABLE 3:** Comparison of blood-brain barrier injury markers between the two groups ($\bar{x}\pm$ s).

Marker	Time point	PE group (*n* = 62)	Non-PE group (*n* = 220)	*t*	*P*
MMP-9 (ng/mL)	D0	185.76 ± 42.39	177.13 ± 38.11	1.536	0.126
	D3	420.72 ± 101.53	284.88 ± 25.14	18.048	<0.001
	D7	385.36 ± 90.92	218.63 ± 52.31	18.471	<0.001
	D14	245.58 ± 60.47	165.27 ± 31.65	14.051	< 0.001
S100β (pg/mL)	D0	185.46 ± 32.33	176.58 ± 32.45	1.905	0.058
	D3	362.01 ± 58.80	225.33 ± 53.27	17.435	<0.001
	D7	337.68 ± 40.52	208.17 ± 51.33	18.315	<0.001
	D14	310.45 ± 72.28	195.46 ± 35.30	17.399	<0.001

**TABLE 4:** Comparison of QAlb levels at D3 between the two groups ($\bar{x}\pm$ s).

Group	QAlb( × 10^–3^)
PE group (*n* = 22)	14.73 ± 3.32
Non-PE group (*n* = 28)	11.99 ± 2.61
*t*	3.269
*P*	0.002

### 3.5 GEE analysis of temporal trajectories of each indicator

For TNF-α, MCP-1, and IL-6, levels rose significantly at D3 and D7 relative to D0 (all *P* < 0.001), followed by a significant decline at D14 (*P* < 0.05). In contrast, MMP-9 and S100β also showed significant increases at D3 and D7 (all *P* < 0.001), but remained above baseline at D14 (*P* < 0.05) (see Table 5).

**TABLE 5:** GEE analysis of temporal trajectories of each indicator.

Indicator	Effect term	β	SE	Wald χ^2^	*P*	95%CI
TNF-α	D3	1.861	0.282	44.12	< 0.001	1.311∼2.412
	D7	0.955	0.223	18.65	< 0.001	0.524∼1.388
	D14	−0.524	0.192	7.49	0.006	−0.897∼−0.155
IL-6	D3	2.561	0.355	53.48	< 0.001	1.871∼3.252
	D7	1.327	0.286	22.22	< 0.001	0.774∼1.876
	D14	−0.684	0.229	9.55	0.002	−1.117∼−0.255
MCP-1	D3	2.187	0.328	46.42	< 0.001	1.554∼2.815
	D7	1.054	0.255	17.64	< 0.001	0.562∼1.545
	D14	−0.481	0.23	5.76	0.016	−0.874∼−0.095
MMP-9	D3	1.524	0.262	34.16	< 0.001	1.014∼2.035
	D7	1.357	0.245	31.64	< 0.001	0.887∼1.829
	D14	0.424	0.183	5.44	0.020	0.077∼0.773
S100β	D3	1.784	0.282	40.42	< 0.001	1.231∼2.332
	D7	1.521	0.257	36.97	< 0.001	1.035∼2.013
	D14	0.568	0.22	7.84	0.005	0.171∼0.956

D0 served as the reference time point for all indicators.

### 3.6 Correlation analysis

**FIGURE 2:**
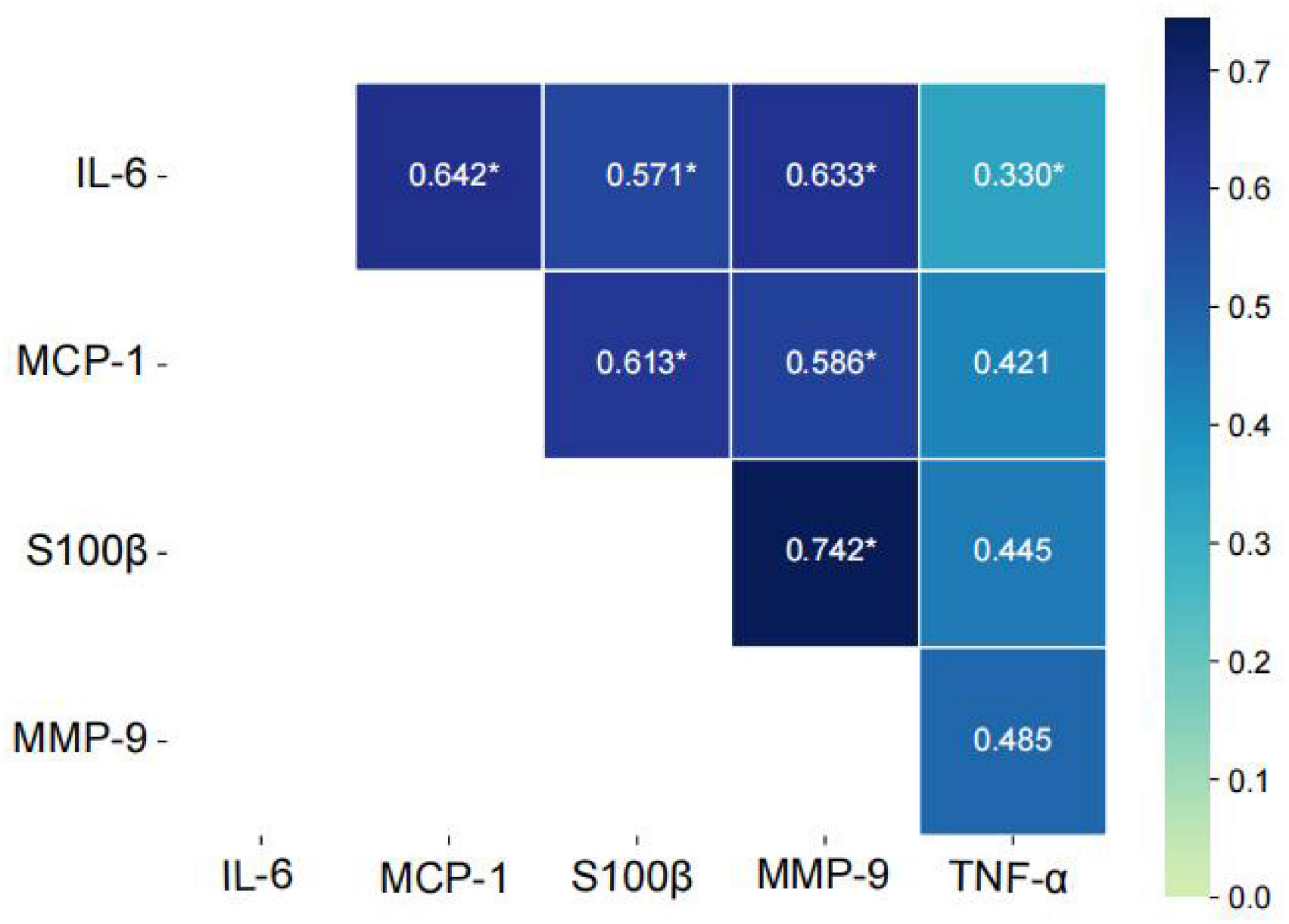
Pearson correlation analysis among indicators. *P < 0.05.

Pearson analysis revealed significant positive associations between IL-6 and S100β (*r* = 0.571), MMP-9 (*r* = 0.633), and TNF-α (*r* = 0.330) (all *P* < 0.05). Similarly, MCP-1 exhibited significant positive correlations with S100β (*r* = 0.613) and MMP-9 (*r* = 0.586) (both *P* < 0.05). Notably, a strong positive correlation was identified between S100β and MMP-9 (*r* = 0.742, *P* < 0.01) (see Figure 2).

### 3.7 Mediation analysis

In the constructed mediation model, Equation 1 showed that IL-6 and MCP-1 had positive predictive effects on MMP-9/S100β. Equation 2 showed that IL-6 and MCP-1 had total effects on PE occurrence. After including the mediator in Equation 3, MMP-9/S100β had a significant effect on PE occurrence, while the direct effects of IL-6 and MCP-1 were both reduced to non-significance (see Table 6).

**TABLE 6:** Mediation model analysis.

Variable	Equation 1 (MMP-9/S100β)			Equation 2 (PE occurrence, total effect)			Equation 3 (PE occurrence, direct effect)		
	β	*t*	*P*	β	*t*	*P*	β	*t*	*P*
IL-6	0.523	8.452	< 0.001	0.451	6.328	< 0.001	0.115	1.533	0.128
MCP-1	0.486	7.924	< 0.001	0.428	6.015	< 0.001	0.116	1.547	0.151
MMP-9/S100β	–	–	–	–	–	–	0.642	9.856	< 0.001
*R* ^2^		0.38			0.372			0.526	
*F*		42.195	< 0.001		38.724	< 0.001		58.632	< 0.001

Equation 1 uses MMP-9/S100β as the dependent variable; Equation 2 uses PE occurrence as the dependent variable to test the total effect; Equation 3 uses PE occurrence as the dependent variable with both independent variables and the mediator included to test the direct effect.

Bootstrap-based mediation analysis indicated that MMP-9/S100β mediated the majority of the total effects of IL-6 and MCP-1 on PE occurrence, with indirect effect estimates of 0.336 (74.50%) and 0.312 (72.90%), respectively. In contrast, the direct effects of both inflammatory markers were non-significant (both *P* > 0.05) (see Table 7).

**TABLE 7:** Bootstrap analysis of mediation effects on PE occurrence path.

Variable		Effect size	Boot SE	Boot 95% CI	Proportion
IL-6 → MMP-9/S100β→ PE	Total effect	0.451	–	–	100%
	Direct effect	0.115	0.075	−0.032 ∼ 0.262	25.50%
	Mediation effect	0.336	0.062	0.214 ∼ 0.458	74.50%
MCP-1 → MMP-9/S100β→ PE	Total effect	0.428	–	–	100.00%
	Direct effect	0.108	0.075	−0.039 ∼ 0.255	27.10%
	Mediation effect	0.312	0.058	0.198 ∼ 0.426	72.90%

### 3.8 ROC analysis

ROC analysis showed that the AUCs of MMP-9, S100β, MMP-9 + S100β, and MMP-9 + S100β + IL-6 for diagnosing PE were 0.811, 0.793, 0.864, and 0.882, respectively, while the AUC of the MMP-9 + S100β + MCP-1 combined model was 0.901. After bootstrap internal validation (1,000 resamples), the calibrated AUC of the MMP-9 + S100β + MCP-1 combined model was 0.887 (95% CI: 0.842–0.925), with an optimism-corrected bias of only 0.014, suggesting a low risk of overfitting. Ten-fold cross-validation showed a mean AUC of 0.883 (SD = 0.031), further supporting good internal consistency and generalizability of the model (see Table 8).

**TABLE 8:** Early diagnostic performance of individual indicators and combined models for PE.

Model/ indicator	AUC	95% CI	*P*	Cutoff	Sensitivity (%)	Specificity (%)	Youden index
MMP-9(D3)	0.811	0.755∼0.871	< 0.001	310.5 ng/mL	78.210	72.505	0.507
S100β(D3)	0.793	0.734∼0.853	< 0.001	265.8 pg/mL	75.403	70.821	0.462
MMP-9 + S100β	0.864	0.812∼0.911	< 0.001	–	81.221	78.636	0.598
MMP-9 + S100β + MCP-1	0.901	0.863∼0.944	< 0.001	–	85.605	82.31	0.679
MMP-9 + S100β + IL-6	0.882	0.845∼0.924	< 0.001	–	83.445	80.101	0.635

### 3.9 Comparison of clinical outcomes

The length of hospital stay in the PE group (28.66 ± 6.43 days) and the 28-day mortality rate (41.94%) were both higher than those in the non-PE group (*P* < 0.05) (see Table 9).

**TABLE 9:** Comparison of clinical outcomes between the two groups [$\bar{x}\pm$ s, n(%)].

Outcome	PE group (*n* = 62)	Non-PE group (*n* = 220)	*t/χ* ^2^	*P*
Hospital stay (days)	28.66 ± 6.43	18.32 ± 4.75	13.929	< 0.001
28-day mortality	26 (41.94)	39 (17.73)	15.981	< 0.001

## 4 Discussion

Although continuous progress has been made in intensive care treatment, the mortality rate of SAP remains high, reaching 30% (Wang X. et al., 2025). PE, as a common severe neurological complication of SAP, mainly manifests as consciousness disorders, disorientation, restlessness, hallucinations, and other neurobehavioral abnormalities (Albacete Ródenas et al., 2022). The pathogenesis of PE is centrally driven by blood-brain barrier compromise, which facilitates the translocation of T lymphocytes and pro-inflammatory mediators from the systemic circulation into the cerebral parenchyma, thereby promoting cerebral edema and neuronal injury (Wang X. et al., 2025). Therefore, in this study, by detecting BBB damage markers and inflammatory markers in the peripheral blood of SAP patients, the application value of these indicators in the early diagnosis of PE was systematically evaluated.

Among the 282 patients with SAP in this study, 62 cases developed PE, with an incidence rate of 21.99%, which was significantly higher than the 11% reported by Meng et al. (2023). This might be due to the differences in baseline characteristics of the populations included in the two studies. This study mainly included SAP patients with an APACHE II score of ≥ 8 points or a CTSI score of ≥ 7 points or persistent organ dysfunction for more than 48 h. The severity of the disease was higher, and the incidence of PE also significantly increased. Comparing the general data of patients in the PE group and the non-PE group revealed that the APACHE II score, CTSI score, and the proportion of persistent organ dysfunction for more than 48 h in the PE group were significantly higher than those in the non-PE group. This further indicates that the occurrence of PE is closely related to the severity of SAP.

By comparing the levels of peripheral inflammatory markers and BBB damage markers in the two groups of patients, it was found that TNF-α, MCP-1, and IL-6 in the PE group were already higher than those in the non-PE group at D0, reached their peak at D3, and remained significantly higher than the non-PE group at D7 and D14. However, MMP-9 and S100β in the PE group showed no significant difference from the non-PE group at D0, but began to significantly increase at D3, maintained a high level at D7, and declined at D14 but remained significantly higher than in the non-PE group. It can be seen that the occurrence of PE is based on the inflammatory background of SAP, and subsequently leads to BBB structure and function damage. The possible reason is that in the early stage of SAP, pancreatic acinar cells are activated by stress or DAMPs stimulation and activate the NF-κB signaling pathway, thereby upregulating the expression of pro-inflammatory cytokines, chemokines, and adhesion molecules involved in the recruitment/migration of leukocytes, and activating the NLRP3 inflammasome to further release DAMPs, forming a cascade amplification of inflammatory signals (Lee et al., 2024). These inflammatory signals promote the aggregation of neutrophils and monocytes to the pancreatic injury site, and the infiltrated leukocytes release more cytokines and aggravate local tissue damage, ultimately leading to extensive pancreatic parenchymal necrosis and systemic inflammatory response syndrome. Under the continuous effect of systemic inflammation, IL-6, which is highly expressed in the peripheral blood, downregulates the expression of tight junction proteins through the JAK/STAT3 pathway, significantly increasing the permeability of the BBB (Kang and Kishimoto, 2021). At the same time, IL-6 can act on vascular endothelial cells to promote the high expression of MCP-1, which directly binds to CCR2 on the vascular endothelial cell membrane, inducing human vascular endothelial cell apoptosis, further exacerbating the structural damage of the BBB (Wang C. et al., 2025), and increasing the level of S100β. After the leukocytes cross the basement membrane of the vascular endothelium, they usually remain in the vascular perivascular space and stay there for a long time. They need to pass through the focal MMP-9-mediated structure of the glial layer to further cross the glial layer and enter the brain parenchyma (Galea, 2021); while the continuously high expression of MMP-9 in SAP state can accelerate this process, allowing a large number of activated leukocytes and inflammatory mediators to break through the BBB and enter the central nervous system, activating microglia and inducing local neuroinflammatory cascade reactions, ultimately leading to neuronal dysfunction and apoptosis. Sternby et al. (2021) pointed out that the levels of TNF-α and IL-6 measured in SAP patients within 24 h after admission were significantly higher than those in patients with moderate AP, and further increased at 25–48 h and remained higher than the moderate AP group, which is similar to the results of this study. It should be noted that this study only had 50 patients who agreed to undergo lumbar puncture, the sample size was relatively small, and only the QAlb level at D3 time point was measured, and the dynamic tracking of QAlb at different time points was not conducted. However, the QAlb in the PE group was significantly higher than that in the non-PE group at D3, which was consistent with the significant increasing trends of MMP-9 and S100β during the same period, further confirming the increase in BBB permeability in PE patients.

Further GEE analysis of the temporal trajectories of each indicator showed that TNF-α, IL-6, and MCP-1 increased at D3 and D7 compared with D0, and significantly decreased at D14 compared with D0. MMP-9 and S100β significantly increased at D3, D7, and D14 compared with D0. The GEE model can more accurately reflect the dynamic changes of each indicator over time (Paragomi et al., 2021). The trend of “first rising and then falling” for inflammatory factors can be observed. The peripheral inflammatory response related to SAP-related PE reaches its peak in the early stage of the disease, and then gradually subsides as the condition is controlled and the body compensates; this suggests that anti-inflammatory treatment is most effective before the peak at D3, and dynamic monitoring of inflammatory marker changes can guide the timing and intensity of anti-inflammatory treatment. The BBB damage marker shows a trend of “continuous increase—slow decline but still higher than the baseline,” indicating that once the structure of the BBB is damaged, the repair process is much slower than the fading of inflammatory factors; this suggests that the risk of PE does not resolve simultaneously with the resolution of peripheral inflammation, and continuous monitoring of BBB markers is helpful for evaluating the risk window of central nervous system involvement, thereby providing a basis for the phased management of PE. Paragomi et al. (2022) confirmed that the Δ modified pancreatitis activity score in patients with severe AP decreased significantly at 24, 48, and 72 h compared to those with mild to moderate AP (*P* < 0.001), which is consistent with the results of this study, indicating that severe patients have a more persistent inflammatory state and BBB structure damage, which in turn leads to a prolonged pathological physiological injury.

The Pearson analysis showed that S100β was strongly positively correlated with MMP-9. MMP-9 is a protease that participates in the degradation of the extracellular matrix. An increase in its level will disrupt the components of the extracellular matrix, damage the integrity of the endothelium, and thereby increase the permeability of the blood-brain barrier (Wu and Jin, 2025). S100β, as an astrocyte-derived calcium-binding protein and a specific marker of glial cells, is released into the peripheral blood in large quantities after BBB disruption (Duan et al., 2021). It should be noted that because TNF-α showed only a weak correlation with IL-6 in this study, it was not included in the subsequent mediation analysis.

Bootstrap mediation analysis revealed that IL-6 and MCP-1 exerted significant indirect effects on PE development via the MMP-9/S100β axis, with mediation effect sizes of 0.336 and 0.312, accounting for 74.50 and 72.90% of the total effects, respectively; the direct effects were not statistically significant, indicating that MMP-9/S100β serves as a complete mediator between peripheral inflammation and PE onset. Intasai et al. (2025) confirmed that MCP-1, by binding to CCR2, guides monocytes to migrate toward the source of chemokines, thereby promoting the aggregation of white blood cells to the inflammatory lesion. Under the SAP context, the highly expressed MCP-1 can continuously recruit monocytes to the vascular wall and tissue injury sites through CCR2, releasing a large amount of inflammatory mediators (Intasai et al., 2025); the high level of IL-6 can upregulate the expression of MMP-9 by activating the JNK/JUN signaling pathway, thereby degrading tight junction proteins and destroying the integrity of the BBB, thereby promoting the release of S100β into the blood (Hu et al., 2025); S100β itself is a neurotoxin, which can induce neuronal apoptosis and damage the survival and function of neurons; at the same time, activating microglia cells triggers central inflammatory responses, further exacerbating nerve damage and promoting the progression of PE (Duan et al., 2021).

The ROC analysis showed that the AUC values for diagnosing PE by MMP-9, S100β, MMP-9 + S100β, and MMP-9 + S100β + IL-6 were 0.811, 0.793, 0.864, and 0.882, respectively. The combined model of MMP-9 + S100β + MCP-1 had an AUC of 0.901, which had the highest predictive efficacy. Bootstrap internal validation and 10-fold cross-validation indicated that the combined model of MMP-9 + S100β + MCP-1 had good internal consistency and generalization potential. This suggests that the combined model of MMP-9 + S100β + MCP-1 can identify high-risk patients with PE at an early stage. In terms of clinical outcomes, the length of hospital stay in the PE group was significantly longer than that in the non-PE group, and the 28-day mortality rate was significantly higher than that in the non-PE group, indicating that PE not only prolongs the hospital stay but also poses a serious threat to the short-term survival of patients. These findings collectively suggest that the combination of MMP-9, S100β, and MCP-1 biomarkers screened and validated in this study is expected to provide an objective basis for the early warning and stratified management of PE. Clinically, anti-inflammatory intervention can be initiated early for high-risk PE patients, and the BBB damage biomarkers can be continuously monitored to assess the risk window of central involvement, thereby improving patient prognosis.

In conclusion, the MMP-9 + S100β + MCP-1 combined model can be used as a detection tool for early identification of patients at high risk of PE in clinical practice. Moreover, this study verified the causal chain of inflammation-induced BBB damage leading to brain injury in PE through time series analysis and mediation effect, providing a reference for targeted MMP-9-based prevention strategies for PE. However, this study was designed as a single-center study and lacked external independent validation, which has certain limitations. Further multi-center research is needed to further verify these findings.

## Data Availability

The raw data supporting the conclusions of this article will be made available by the authors, without undue reservation.
